# The HOSENG trial – Effect of the provision of oral self-testing for absent and refusing individuals during a door-to-door HIV-testing campaign on testing coverage: protocol of a cluster-randomized clinical trial in rural Lesotho

**DOI:** 10.1186/s13063-019-3469-2

**Published:** 2019-08-13

**Authors:** Alain Amstutz, Thabo Ishmael Lejone, Lefu Khesa, Josephine Muhairwe, Bienvenu Lengo Nsakala, Katleho Tlali, Moniek Bresser, Fiona Vanobberghen, Mathebe Kopo, Mpho Kao, Thomas Klimkait, Manuel Battegay, Niklaus Daniel Labhardt, Tracy Renée Glass

**Affiliations:** 1 Clinical Research Unit, Department of Medicine, Swiss Tropical and Public Health Institute, Socinstrasse 57, 4051 Basel, Switzerland; 2 University of Basel, 4051 Basel, Switzerland; 3 Division of Infectious Diseases and Hospital Epidemiology, University Hospital Basel, 4051 Basel, Switzerland; 4 SolidarMed, Swiss Organization for Health in Africa, Maseru West, Lesotho; 5 Butha-Buthe Government Hospital, Butha-Buthe, Lesotho; 6 Department of Biomedicine, University of Basel, 4051 Basel, Switzerland

**Keywords:** HIV, Cluster-randomized controlled trial, Village health worker, Community health worker, Community-based, Self-testing, HIV-testing coverage, Lesotho, Southern Africa, Implementation research

## Abstract

**Background:**

HIV-testing coverage remains below the targeted 90% despite efforts and resources invested. Home-based HIV-testing is a key approach endorsed by the World Health Organization (WHO), especially to reach individuals who might not seek testing otherwise. Although acceptance of testing during such campaigns is high, coverage remains low due to absent household members. This cluster-randomized trial aims to assess increase in testing coverage using oral HIV self-testing (HIVST) among individuals who are absent or decline testing during home-based HIV-testing.

**Methods:**

The HOSENG (HOme-based SElf-testiNG) trial is a cluster-randomized, parallel-group, superiority trial in two districts of Lesotho, Southern Africa. Clusters are stratified by district, village size, and village access to the nearest health facility. Cluster eligibility criteria include: village is in catchment area of one of the study facilities, village authority provides consent, and village has a registered, capable, and consenting village health worker (VHW). In intervention clusters, HIV self-tests are provided for eligible household members who are absent or decline HIV-testing in the presence of the campaign team. In control clusters, standard of care for absent and refusing individuals applies, i.e., referral to a health facility. The primary outcome is HIV-testing coverage among individuals aged 12 years or older within 120 days after enrollment. Secondary objectives include HIV-testing coverage among other age groups, and uptake of the different testing modalities. Statistical analyses will be conducted and reported in line with CONSORT guidelines. The HOSENG trial is linked to the VIBRA (Village-Based Refill of ART) trial. Together, they constitute the GET ON (GETting tOwards Ninety) research project.

**Discussion:**

The HOSENG trial tests whether oral HIVST may be an add-on during door-to-door testing campaigns towards achieving optimal testing coverage. The provision of oral self-test kits, followed up by VHWs, requires little additional human resources, finances and logistics. If cost-effective, this approach should inform home-based HIV-testing policies not only in Lesotho, but in similar high-prevalence settings.

**Trial registration:**

ClinicalTrials.gov, (ID: NCT03598686). Registered on 25 July 2018. More information is available at www.getonproject.wordpress.com.

**Electronic supplementary material:**

The online version of this article (10.1186/s13063-019-3469-2) contains supplementary material, which is available to authorized users.

## Background

The United Nations Programme on HIV/AIDS (UNAIDS) has shown a way forward in controlling and finally ending the deadly acquired immune deficiency syndrome (AIDS) epidemic by launching the 90-90-90 strategy [1]. The first target is to ensure that 90% of all people living with human immune deficiency virus (HIV) are aware of their status. The second target is that 90% of those diagnosed receive sustained antiretroviral therapy (ART). And the third target stands for 90% of those receiving ART achieving viral suppression. Globally, in 2017, progress towards the first UNAIDS target, i.e., that 90% of people living with HIV are aware of their status, was lower than other stages of the HIV care cascade [2]. The most recent data from Eastern and Southern Africa show that the percentage of people living with HIV who know their HIV status has steadily improved in the last few years, currently at a level of 81% (64–95%), but still leaving a gap of approximately 1.7 million HIV-positive people to reach the target of 90% knowing their HIV status [3, 4]. In order to reach the first UNAIDS target, home-/community-based testing, i.e., HIV-testing close to where people live or work, is a key strategy endorsed by the World Health Organization (WHO) [5, 6]. Many studies from Southern Africa, including Lesotho, have shown that home-based HIV-testing is highly promising for closing the crucial gap to achieve the first 90 [7]. Yet, it is critical to distinguish between the principal acceptance (uptake) of HIV-testing and the HIV-testing coverage. While in most cases the former is above 90%, the coverage often remains below 90% due to the household members who are absent at the time of the campaign, mainly men and young adults [8].

HIV self-testing (HIVST) has proved to be an accurate diagnostic tool that increases uptake of HIV-testing, facilitates linkage to care among target populations, and may be more cost-effective than provider-delivered HIV-testing [9–17]. Thus, WHO strongly endorses oral HIVST [18]. In a WHO review, 16 countries reported having a policy supportive of HIVST, including Lesotho [19, 20].

However, to date only limited data are available, assessing the effect of oral HIVST in increasing HIV-testing coverage. This randomized controlled trial thus aims to determine the added effect of distributing oral HIVST to individuals absent or refusing to test during a home-based HIV-testing campaign on HIV-testing coverage.

## Methods

### Setting

The HOSENG (HOme-based SElf-testiNG) trial will be conducted in the districts of Butha-Buthe and Mokhotlong, in Northern Lesotho, Southern Africa, in the catchment areas of 22 health facilities. Both districts are characterized by mostly rural settings with an estimated population of 220,000, mainly subsistence farmers and mine workers as well as construction or domestic laborers who work in neighboring South Africa. Each district has only one mid-size town: Butha-Buthe with approximately 25,000 inhabitants, and Mokhotlong with approximately 10,000 inhabitants. The remaining population lives in villages scattered over a mountainous area of 5842 km^2^. According to the recent household-based national survey from 2016 to 2017 the adult HIV prevalence is 17.8% in Butha-Buthe and 26.1% in Mokhotlong [21].

### Design

The HOSENG trial is a cluster-randomized controlled, superiority trial in a resource-limited setting. The rationale for a cluster-randomized design at village level is (1) the reliance of the trial on the village health workers (VHWs) and (2) the high risk of cross-contamination between the study arms if randomization would be done at the individual or household level. The HOSENG trial, with its home-based HIV-testing campaign, provides a recruitment platform for another trial, the VIBRA (Village-Based Refill of ART) trial [22], and thus they are based on the same cluster-randomization and run in parallel. Together, HOSENG and VIBRA [22] constitute the GET ON (GETting tOwards Ninety) research project. To ensure a balance in the exposure to the HOSENG intervention in the two arms, the clusters (villages) will be randomized into four potential groups in a 1:1:1:1 allocation.

### Cluster sampling and randomization

**Fig. 1:**
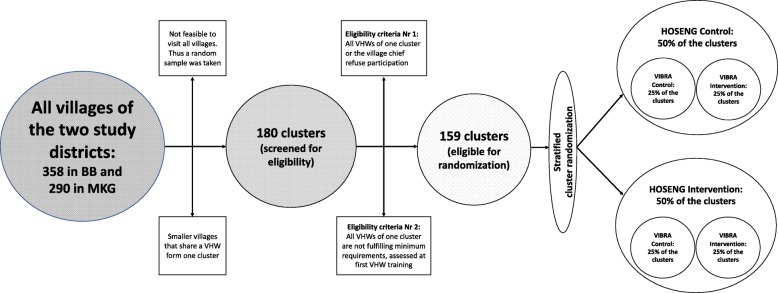
Cluster sampling and randomization. Abbreviations: *BB* Butha-Buthe, *MKG* Mokhotlong, *VHW* village health worker

Figure 1 summarizes the cluster sampling and randomization process. A list of all villages with their corresponding VHWs in the study districts was provided by the Ministry of Health and the local District Health Management Teams. Two local members of the research team cross-checked the village list for accuracy. They defined the village size (≥ 30 versus < 30 households) and access to the nearest health facility (easy versus hard to reach, defined by needing to cross a mountain or river or > 10 km away from health facility) by contacting the relevant VHW coordinators. Each village was considered a cluster, except villages which do not have their own VHW and, therefore, form one cluster with neighboring villages served by the same VHW. It is not feasible to visit all clusters (358 villages in Butha-Buthe and 290 villages in Mokhotlong) nor to include more than two VHWs per cluster. Therefore, a random sample of 180 clusters, stratified by district, village size, and access, was taken. If a cluster had more than two VHWs, randomly two VHWs were selected. After excluding clusters who did not meet the eligibility criteria (see next section), the remaining 159 clusters were randomized into the four groups in a 1:1:1:1 allocation ratio with block sizes of 4, stratified by the same stratification factors. All random sampling processes as well as the randomization were performed by an independent statistician. The first 103 clusters will be provided to the research team and will all be enrolled. If needed, further clusters will be released from the randomization list.

### Eligibility criteria

Table 1 outlines the eligibility criteria for clusters and households. No individual eligibility criteria apply for the HOSENG trial as HIV-testing coverage is a population-level outcome including every individual in the surveyed area.

**Table 1:** Cluster and household eligibility criteria for the HOme-based SElftestiNG (HOSENG) trial

	Inclusion criteria	Exclusion criteria
Cluster eligibility		
1	The cluster is rural and clearly confined to the catchment area of one of the study clinics a. Note: 1 cluster usually consists of 1 village, but could include several small villages if serviced by the same VHW	The village authority (= village chief) is opposed to trial participation
2	The cluster has at least 1 registered VHW who is willing to participate and fulfills the following criteria: a. is at least 18 years of age b. has adequate reading and writing skills c. successfully passes the training assessment evaluated by a local person independent to the research project and the research team, by checking if the VHW: 1) is able to fill in the assessment (ticking boxes, writing in correct fields) 2) is able to give an adequate answer regarding the open question, which implies adequate reading and writing skills and a basic logical thinking	The VHW is not willing to attend the training or opposed to trial participation or not fulfilling the criteria mentioned in inclusion criteria a. Note: if a cluster entails several VHWs, then the cluster can still participate if there is at least 1 VHW in the cluster who is willing to participate and fulfills the criteria
Household eligibility		
1	Signed informed consent from household head or representative aged 18 years or older	No signed informed consent from household head or representative aged 18 years or older

*VHW* village health worker

Before randomization, all VHWs from the 180 randomly selected villages attend a 1-day refresher training focusing on HIV-testing and counseling (HTC). Every VHW takes a short pre- and post-training assessment. The assessment includes questions about (1) HIV knowledge and HIV stigma using a validated questionnaire [23], (2) HIV-testing knowledge, and (3) one open question (“What are important qualities of a counselor?”). The assessment will be used to evaluate the baseline HIV/AIDS-related knowledge and stigma of the involved VHWs and to evaluate their eligibility for participation in the trial (Table 1).

Prior to trial start, the research team met with all relevant village chief councils in order to inform them about the trial and to obtain their consent.

### Procedure

**Fig. 2:**
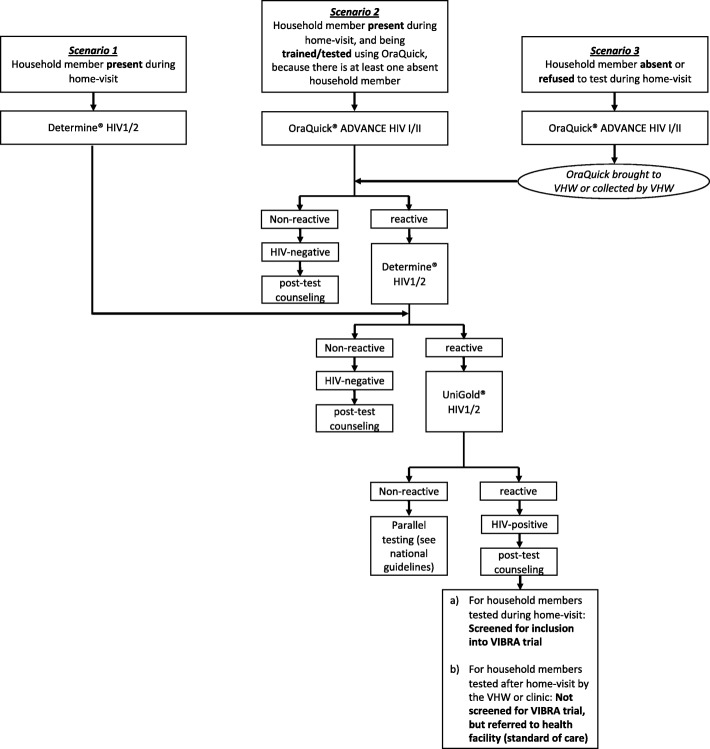
HIV-testing scenarios in HOme-based SElftestiNG (HOSENG) trial. Scenarios 2 and 3 only occur in the HOSENG intervention clusters

Two to three specifically trained teams, each consisting of five to six lay-counselors, one campaign organizer and one supervising study nurse will visit all households in selected villages, going from door to door. The teams will propose HTC and multi-disease screenings and prevention. The population of the selected areas are informed about the campaign beforehand by their village chief. Specifically, the campaign team will arrive in the village in the morning, meet with the village chief and then start systematically visiting all households in the pre-defined area by going from door to door. At the household, the teams will proceed as follows:

1. The counselor will introduce him- or herself and explain the purpose of the testing/screening campaign
2. The counselor will ask the head of household or their representative (aged 18 years and older) for written informed consent to the testing/screening campaign, and to obtaining data about present and absent household members for study purpose
  - If the head of household or the representative refuses their household participation, the team will leave the house, record the refusal reason, and proceed to the next household
3. The counselor will assess the total number of present and absent household members
  - Definition of household member: (1) is acknowledged by the household head or the representative as part of the household and (2) sleeps in the household regularly (at least once a month), and (3) if absent during the campaign: returns to the household no later than 3 months after the date of the home visit
4. The counselor will provide information about HIV and testing, prevention aspects, and the other disease screenings (see below)
5. The counselor will assess the HIV status of all household members and screen the patient’s health booklets (“*bukana*”)
6. Household members who are eligible for and consent to testing (by filling in the Lesotho national informed consent form for HIV-testing) undergo HIV-testing by the counselor according to specific cluster-arm allocation procedure and according to national HIV-testing guidelines [20]. For absent or declining household members the specific procedures differ between the two cluster arms and are described in detail below in the section on intervention clusters. Figure 2 presents all possible HIV-testing scenarios during the campaign
  - Once the HIV test is done, the counselor documents the result in the patient’s health booklet and provides post-test counseling. If the HIV test is confirmed positive, the counselor contacts the study nurse, who then provides further counseling and assesses the participant for enrollment into the inter-linked follow-up study (VIBRA trial [22])

**Fig. 3:**
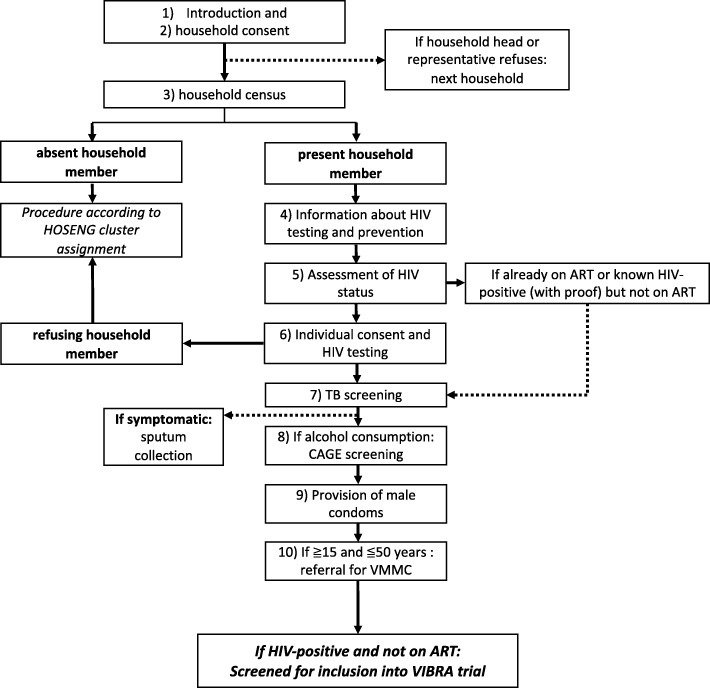
Algorithm of the human immune deficiency virus (HIV) and multi-disease screening/prevention campaign

The HIV-testing campaign is combined with additional multi-disease screening. The campaign screens for tuberculosis (TB) according to national guidelines using the clinical symptom screening tool [24]. If TB is suspected, one sputum sample is collected on the spot, transported to the health facility the same day by the campaign team for testing with GeneXpert. Another sputum bottle for collecting a morning sample is left behind, the individual is instructed on how to use it and a follow-up plan for collection (i.e., collection by VHW) is agreed upon. Further campaign services include alcohol abuse screening using the CAGE questionnaire [25], information and referral of men aged 15–50 years (HIV-negative and -positive due to stigma reasons) for voluntary medical male circumcision (VMMC) according to WHO recommendations [26], and promotion and provision of male condoms. Figure 3 presents the overview of the HIV and multi-disease campaign and its algorithm.

#### Intervention clusters

**Fig. 4:**
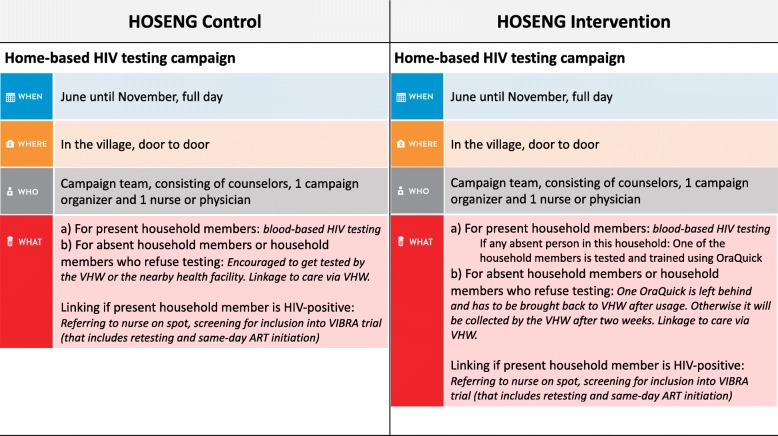
Description of the procedure in the HOme-based SElftestiNG (HOSENG) intervention and control clusters. Mobilizing: through village chief before the campaign date, and through the campaign team on the day of the campaign by going from door to door. Further services include: screening for tuberculosis, screening for alcohol abuse, voluntary medical male circumcision and condom provision

**Fig. 5:**
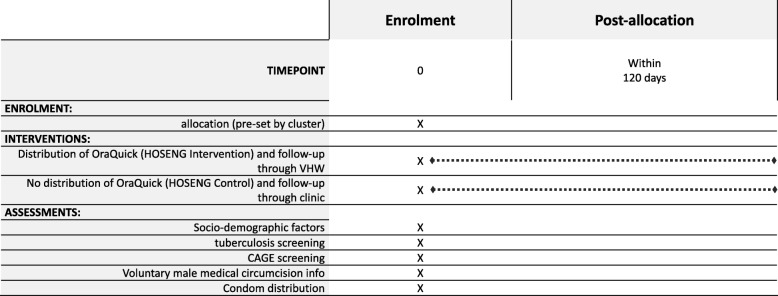
Standard Protocol Items; Recommendations for Interventional Trials (SPIRIT) flow diagram of the HOme-based SElftestiNG (HOSENG) trial

Figure 4 summarizes the procedures in the HOSENG clusters and Fig. 5 the Standard Protocol Items: Recommendations for Interventional Trials (SPIRIT) flow diagram. In the intervention clusters, the campaign team will leave an oral HIVST kit (OraQuick® ADVANCE HIV I/II, second generation serology assay with a sensitivity of > 93%, a specificity of > 99 [27–30]) for every household member aged 12 years or older who is absent or declined HIV-testing on the day of the campaign. We chose 12 years as the threshold because adolescents are an important risk group and this is the legal age for providing HIV-testing consent in Lesotho [24]. The household can refuse to have an oral HIVST left behind for their absent household members. The oral HIVST kit is prepacked, includes a written and pictoral instruction for use in the local language, Sesotho, and the package is labelled with a written request to consult the VHW within 2 weeks after use of the test – irrespective of the result. In cases where more than one trained VHW serves the village, the household will be asked for the VHW of choice. The team will label the kit with the name of the absent member before dispensation.

One household member – the one with the closest relation to the absent person(s) – will be tested and trained using the oral HIVST. All other present household members will be tested using the standard blood-based point-of-care HIV test according to Lesotho national guidelines [20].

The VHWs in the intervention custers will receive a list of all household members for whom an oral HIVST was dispensed. The VHW will visit all households 2–4 weeks after the campaign to collect the oral HIVST in case the oral HIVST is not returned to the VHW. If an oral HIVST is reactive, the VHW will either provide further blood-based testing on the spot or organize referral to the nearby health facility for confirmatory testing. All VHWs from intervention clusters will receive a second extensive training session about oral HIVST, handling disclosure and stigma, and data entering on paper-based study forms and the patient’s health booklet.

#### Control clusters

Following standard of care during home-based HIV-testing, every absent household member or those declining HIV-testing will be encouraged to get an HIV test done by the VHW or the nearby health facility. The research team will screen the registers at the health facilities in order to assess if these individuals came for testing in the timeframe of the primary endpoint window.

### Endpoints

The primary endpoint is HIV-testing coverage among individuals aged 12 years or older in the surveyed area within 120 days after the home visit, defined as the proportion of all individuals aged 12 years or older living in a household of the surveyed area with a confirmed HIV test result.

We define a confirmed HIV-negative result as either being tested HIV-negative as per the algorithm defined in Fig. 2, or being tested HIV-negative within the last 4 weeks with proof of documentation (i.e., documentation in patient’s health booklets). We define a confirmed HIV-positive result as (1) tested HIV-positive as per the algorithm defined in Fig. 2, or (2) being tested HIV-positive but not yet on ART with proof of documentation, or (3) being on ART with proof of documentation.

The rationale for the time point of 120 days is that we leave oral HIVST kits at the households for all absent household members aged 12 years or older who return within a maximum of 3 months (see definition of household member above in the “Procedure” section). An interval of 120 days after home visit allows sufficient time for absent members to return to their households, conduct self-testing, and be followed up by the VHW.

The secondary endpoints are outlined in Table 2.

**Table 2:** Secondary endpoints of the HOme-based SElftestiNG (HOSENG) trial

	Endpoint	Definition	Time point following enrollment
1	Testing coverage irrespective of age	Proportion of all individuals living in a household of the surveyed area with a confirmed HIV test result	Within 120 days
2	Blood-based HIV-testing uptake	Proportion of all present individuals living in a household of the surveyed area, being eligible for blood-based HIV-testing and accepting to be tested using blood-based point-of-care HIV test	On the day of the campaign
3	Oral-based HIV-testing uptake	Proportion of all individuals living in a household of the surveyed area for whom an oral HIV self-test (HIVST) was left behind and who performed it	Within 120 days

### Additional research within the project

For the entire GET ON project we will collect cost data, see more details in the VIBRA trial protocol [22] published elsewhere. Specifically, for the HOSENG trial, first, direct costs of the intervention will be assessed: Staff costs (campaign team, VHWs, clinic staff), personnel training costs (VHWs), cost of equipment (HIV tests, consumables, logistics), as well as non-medical costs to the participant (i.e., cost of transportation to the ART service). These data will provide the cost per participant achieving the primary endpoint within 120 days in each cluster arm (“per participant tested cost”). Secondly, a cost-effectiveness analysis will be performed with respect to the primary endpoint. The cost-effectiveness ratio will be presented as incremental cost per additional confirmed HIV test result. Data to assess patient level costs will be collected from a randomly selected sub-sample of study participants from each cluster arm. Costs will be reported in local currencies and US dollars and International dollars. The robustness of results will be tested with probabilistic sensitivity analysis and deriving cost-effectiveness acceptability curves to capture the uncertainty around the probability that the intervention is below the relevant cost-effectiveness thresholds.

A nested study (ADORE study: “ADolescent ORal sElf-testing”) will explore the effectiveness acceptability of oral HIVST among adolescents and young adults with quantitative methods (testing coverage among adolescent and young adults, defined as the proportion of all 12–24-year-old individuals living in a household of the surveyed area with a confirmed HIV test result) and qualitative methods (case-control study). Cases are those who refused testing through oral HIVST and controls are those who accepted testing through oral HIVST. We plan to conduct at least 10 interviews per group, stratified by two pre-defined factors (male vs female; age 12–15 years vs age 16–24 years), following the concept of saturation. Data will be collected by a trained study member, who was part of the HIV-testing campaign, using a piloted interview questionnaire (KoboToolbox; www.kobotoolbox.org), conducted in the local language (Sesotho). Qualitative data will be recorded, transcribed, translated into English and coded and analyzed using the Framework Method [31].

### Data collection and management

The campaign team will capture all data collected during the campaign using a tablet-based application and platform (MACRO, Elsevier). The randomization assignment of the villages is pre-loaded into the program and a unique household identifier is automatically generated. Before leaving the household, the completed questionnaire will be checked for mistakes and completeness. Data from the tablet devices will be uploaded regularly via secure electronic transfer and stored on a secure server at the Swiss Tropical and Public Health Institute (Swiss TPH). After the follow-up period, all confidential information of the study participants (i.e., names) will be deleted from the database and only the anonymous study ID will be kept. The informed consent forms will be stored in a secure way in the headquarter of the study center (SolidarMed Office in Butha-Buthe, Lesotho). Participant files will be maintained in storage for a period of at least 10 years after completion of the trial.

The VHWs enter data for following up the dispensed oral HIVST kits on standardized paper study forms (case report forms) that act as source documents. These data will be collected regularly by the study team and entered into the above-mentioned platform. A study data manager will monitor data quality and completeness on a weekly basis. Queries about the data will be sent to the local principal investigators for follow-up and correction, as needed. Data integrity checks will be written into the database to limit missing fields or the entry of incorrect data. The type of activity that an individual user in the online study database may undertake will be regulated by the privileges associated with their user identification code and password.

### Sample size

The sample size is driven by the VIBRA trial [22]. We assume that to achieve the VIBRA trial [22] sample size (minimum 262 HIV-positive individuals not on ART) by testing about 10,000 individuals in 100 villages, based on a previous trial [32]. According to a previous home-based HIV-testing campaign [33], we estimate an HIV-testing coverage rate of individuals aged 12 years or older in rural villages of 63%. We consider a 15% increase in coverage as relevant from a policy perspective. Using an intra-cluster correlation (ICC) of 0.028, with a sample of 100 clusters, we will require a sample size of 450 in order to have > 90% power to detect an increase in coverage rates to 78% or higher. This corresponds to an increase of 1.17 (variance inflation factor (VIF)) from the 386 individuals required in the absence of clustering. If we vary the ICC for villages and include an additional ICC for clustering at the household level [34], the maximum VIF would be 8.3 corresponding to a maximum sample size of 3204 needed to ensure 90% power to detect a 15% difference, well below the expected 10,000 individuals to be included. Table 3 displays the sample size calculations considering all relevant factors under different scenarios.

**Table 3:** Sample size estimations for the HOme-based SElftestiNG (HOSENG) trial

Average village size	Average household size	ICC (village)	ICC (household)	VIF	Total number of clusters	Total sample size
30	2	0.028	0.05	1.04	100	402
30	2	0.028	0.1	1.07	100	413
30	2	0.05	0.05	1.20	100	462
30	2	0.05	0.1	1.34	100	518
30	2	0.1	0.05	2.95	100	1139
30	2	0.1	0.1	4.4	100	1699
30	4	0.028	0.01	1.06	100	410
30	4	0.028	0.05	1.31	100	507
30	4	0.028	0.1	1.62	100	628
30	4	0.028	0.5	4.12	100	1592
30	4	0.05	0.01	1.09	100	420
30	4	0.05	0.05	1.44	100	556
30	4	0.05	0.1	1.88	100	726
30	4	0.05	0.5	5.4	100	2085
30	4	0.1	0.01	1.15	100	442
30	4	0.1	0.05	1.73	100	668
30	4	0.1	0.1	2.46	100	950
30	4	0.1	0.5	8.3	100	3204

*ICC* intracluster correlation coefficient, *VIF* variance inflation factor

### Analyses

Analyses will be reported following Consolidated Standards of Reporting Trials (CONSORT) guidelines for cluster-randomized trials [35]. Clusters will be set as the unit of randomization (stratified by district, size of village, and village access to the nearest health facility), whereas individuals are set as the unit of analysis. An intention-to-treat set will be used, i.e., all study participants will be evaluated according to arm assignment at randomization. The primary analysis will use multi-level logistic regression models including village and household as random effects to assess the difference between HIV-testing coverage in the intervention versus the control arm. These models will be adjusted for the pre-specified randomization stratification factors and relevant baseline factors (age groups, gender, education status, employment status, HIV-testing history) that may be randomly unbalanced between intervention and control clusters [36, 37].

Baseline characteristics will be presented according to randomized groups; no formal testing will be performed. Categorical variables will be described with absolute and relative frequencies and continuous variables with medians and interquartile ranges. As with the primary analysis, secondary endpoints will be analyzed with multi-level logistic regression models including village and household as random effects. All results will be presented as odds ratios and 95% confidence intervals. The potential effect modification of sociodemographic determinants (age groups, gender, education status, employment status, HIV-testing history) on all endpoints will be assessed by including interaction terms in the models. If the Wald test results for the interaction terms are significant, intervention effects will be presented separately by the levels of these factors. Sensitivity analyses will be conducted in order to provide evidence that the result seen from the primary analysis are robust. In order to assess the reliability of the model fit, we will perform a quadrature check. In case of unreliable model fit, we will use generalized estimating equations to fit our model which will provide population-averaged odds ratios and 95% confidence intervals. However, this model would not allow inclusion of more than one random effect although including the highest level of clustering is suggested to be sufficient [38]. All analyses will be done using Stata (version 14, Stata Corporation, Austin, TX, USA). For all tests, we will use two-sided *p* values with an alpha = 0.05 level of significance.

### Monitoring, auditing, and data safety and monitoring board

At least one external monitoring visit will assess adherence to the approved trial protocol, accuracy of completed study forms, and the electronic dataset. The principal investigator agrees to allow inspectors from regulatory agencies to review records and will assist the inspectors in their duties, if requested.

The HOSENG trial represents implementation research and the oral HIVST is a well-established diagnostic tool. Thus, we do not expect serious adverse effects (SAE) on patients’ health from this intervention. However, for the purpose of this trial, we will try to capture the following SAEs: (1) Death due to any reason (especially within 30 days of a positive HIVST results), (2) Hospitalisation due to self-inflicted injuries within 30 days of a positive HIVST results, and (3) Hospitalisation resulting from violent assault by others (intimate partner violence, assault by family or community members) within 30 days of a positive HIVST result. The campaign team members all have several years of experience in HTC and received an additional study-specific, 1-week training in order to handle adverse events related to testing stigma. A separate, detailed safety monitoring plan will be developed to handle these SAEs in line with Swiss and Basotho ethics regulations. It is not planned to establish a data safety and monitoring board.

## Discussion

HIV-testing constitutes the “front door” to reach the UNAIDS 90-90-90 targets [39]. Currently, UNITAID invests US$23 million into a 4-year research initiative, called STAR, to gather evidence and catalyze the market for HIVST, and eventually improve testing coverage through HIVST in three African countries (Malawi, Zambia, and Zimbabwe) [40]. Whereas the STAR trials assess HIVST on a large scale, the HOSENG trial will focus on the use of oral HIVST during home-based HIV-testing campaigns. As such, its result will be complementary to the findings of the STAR project.

Home-based HIV-testing campaigns have been shown to be very effective in achieving high testing uptake, especially in resource-limited countries [7]. However, one major question is still unanswered: How to reach the absent people during these campaigns in a cost-effective way? The HOSENG trial tests whether oral HIVST may be an effective add-on during door-to-door testing campaigns towards achieving optimal testing coverage. Linkage to further testing and care after usage of oral HIVST will be provided by the VHWs, a trusted and long-standing public-sector cadre. VHW programs exist in all countries of Southern Africa and are being expanded [41]. If cost-effective, the HOSENG approach could easily be scaled up in the region, as the provision of oral self-test kits, followed up by VHWs, requires little additional human resources, finances, and logistics. The HOSENG trial will inform home-based HTC policies not only in Lesotho, but in similar nations in sub-Saharan Africa. However, we will have to closely monitor the linkage after testing and the additional burden of work for the VHWs.

## Trial status and recruitment

The trial has been launched on 26 July 2018 in both study districts. The recruitment time is driven by the VIBRA trial [22], as the HOSENG trial is the main recruitment platform for the VIBRA trial [22]. We assume a recruitment period of 6–8 months.

## Additional file

Additional file 1: Standard Protocol Items: Recommendations for Interventional Trials (SPIRIT) 2013 Checklist: recommended items to address in a clinical trial protocol and related documents*. (PDF 82 kb)

## Data Availability

The datasets used and/or analyzed during the study will be available from the corresponding author on reasonable request. The filled-in SPIRIT checklist was uploaded as Additional file 1.

## Abbreviations

ADORE: ADolescent ORal sElf-testing
ART: Antiretroviral therapy
GET ON: GETting tOwards Ninety
HIV: Human immune deficiency virus
HIVST: HIV self-test/ing
HOSENG: HOme-based SElftestiNG
HTC: HIV-testing and counseling
ICMJE: International Committee of Medical Journal Editors
SAE: Serious adverse events
Swiss TPH: Swiss Tropical and Public Health Institute
TB: Tuberculosis
UNAIDS: United Nations Programme on HIV/AIDS
VHW: Village health worker
VIBRA: Village-Based Refill of ART
VIF: Variance inflation factor
VMMC: Voluntary medical male circumcision
WHO: World Health Organization
